# Fatty acid and cholesterol profiles and hypocholesterolemic, atherogenic, and thrombogenic indices of table eggs in the retail market

**DOI:** 10.1186/s12944-015-0133-z

**Published:** 2015-10-27

**Authors:** Youssef A. Attia, Mohammed A. Al-Harthi, Mohamed A. Korish, Mohamed M. Shiboob

**Affiliations:** Arid Land Agriculture Department, Faculty of Meteorology, Environment and Arid land Agriculture, King Abdulaziz University, Jeddah, Saudi Arabia; Environmental Department, Faculty of Meteorology, Environment and Arid land Agriculture, King Abdulaziz University, Jeddah, Saudi Arabia

**Keywords:** Eggs lipids, Atherogenic, Thrombogenic, Retail market

## Abstract

**Background:**

Eggs are an important source of food due to its favorable effects on human health derived from the protein, fats, minerals, vitamins and bioactive components. We studied the effects of source of eggs in the retail market on fatty acids, lipid profiles and antioxidant status in eggs.

**Methods:**

Eggs from four sources named A, B, C, and D in the retail market were collected to determine fatty acid, total lipid, and cholesterol profiles; hypocholesterolemic, atherogenic and thrombotic indices; antioxidant status (e.g., of malondialdehyde); and total antioxidant capacity in the whole edible parts of eggs (albumen + yolk) and egg yolk. Samples were collected four times and pooled over times to represent 5 and 10 samples per source for determinations of fatty acids and determinations of lipid profiles and antioxidant status, respectively.

**Results:**

Fatty acid, total lipid, and cholesterol profiles; hypocholesterolemic, atherogenic and thrombotic indices; presence of malondialdehyde; and total antioxidant capacity in the whole edible parts of eggs and egg yolk showed significant differences (*P* ≥ 0.05) among different sources of eggs in retail market. Source D showed higher levels of saturated fatty acids (SFA) and linoleic and monounsaturated fatty acid (MUFA)/polyunsaturated fatty acid (PUFA) ratio but lower levels of MUFA and linolenic, arachidonic, eicosapentaeonic (EPA), decohexaenoic (DHA), and total ω9 fatty acids and lower unsaturated fatty acids (UFA)/SFA ratio. Similar trend was shown in fatty acids profiles of the whole edible parts of eggs. On the other hand, total cholesterol, low density lipoprotein (LDL), LDL/high density lipoprotein (HDL) ratio, and atherogenic and thrombogenic indices and total antioxidant capacity of source D were significantly higher than those of other source, but levels of hypocholesterolemic index, and malondialdehyde levels were lower for source D.

**Conclusion:**

Eggs in the retail market in Jeddah city, Saudi Arabia, from May to August 2015 showed a different pattern of fatty acid and cholesterol profiles; hypocholesterolemic, atherogenic, and thrombogenic indices; and antioxidant status, which might reflect the nutritional and husbandry practice of laying hens. This can affect the nutritional values of eggs, and hence, customer benefits, suggesting the need for standardization and quality control based on nutrient index values.

## Background

Eggs are a principle food for human consumption practically for the children and elderly, it is delicious, easy to digest, and contains most of the nutrients needed by human based on recommended daily allowance. It is found on breakfast and dinner tables and is used for enrichment of other human foods [1–4]. Egg yolk is a rich source of both nutritive and non-nutritive compounds important to human health [5]. However, although eggs contain all the necessary nutrients for life, consumption of eggs may negatively affect because of high cholesterol content [1, 6, 7]. Although eggs contain essential proteins, UFA, minerals, and vitamins, it is suggested that egg consumption should be limited due to high cholesterol [5, 8]. It is also believed that increasing consumption of the eggs elevates the cardiovascular threat by increasing levels of blood cholesterol [9]. Concentrations and type of fat/fatty acids consumption were reported to affect cell membrane, tissues, egg-yolk lipid composition, and concentrations of lipoprotein in plasma [10]. Consumption of fatty acids can have a direct effect on stimulate or preclude atherosclerosis and coronary thrombosis due to their effect on blood cholesterol and low density lipoprotein (LDL)–cholesterol concentrations [10]. Accordingly, the atherogenic index has been introduced [11], and eggs with lower SFA/UFA ratio showed low values of atherogenic, thrombogenic, and hypercholesterolemic indices, and they were recommended for a healthy diet [10, 12, 13]. The C14:0 and C16:0 fatty acids are known to be among the most atherogenic, whereas C18:0 is believed to be neutral with respect to atherogenicity but is instead considered to be thrombogenic [14–16]. In contrary, recent evidences suggested there is no direct link between egg consumption and blood cholesterol levels [17, 18]. Fatty acids and cholesterols in eggs are essential components from health and consumption prospective for human particularly in terms of polyunsaturated fatty acid (PUFA) and omega-3 fatty acids consumption. It is well known that hens’ diet, particularly that containing fats/fatty acids, strongly influences egg composition [7, 19–23]. Although there are general belief that eggs in the retail market have similar nutrient profiles and quality, through dietary manipulation, certain fatty acids and several nutrients with important health implication can be affected [1, 7, 24, 25]. Among the different factors affecting egg quality and composition are dietary profile and type of fats [26, 27], breed and strain of layers [6, 28–30], health of birds, environmental conditions and husbandry practice [30–32]. This study was performed to monitor the fatty acids, cholesterol profiles, atherogenic and thrombogenic indices, presence of malondialdehyde, and total antioxidant capacity of the eggs from four sources in the retail market in Jeddah city, Saudi Arabia, and their capability to fulfill RDA.

## Results

### Fatty acid profiles of the whole edible parts (albumen + yolk)

Results for fatty acid content of the whole edible parts of eggs are presented in Table 1. Differences among various sources of eggs were significant in terms of majority of fatty acids, except for capric, lauric, myristic, arachidic, myristoleic, palmitoleic, erucic, and linoleic acids and decohexaenoic acid (DHA). In addition, differences in PUFA and total ω3 content and ω6/ω3 fatty acid ratio were not significant. Eggs from source D had greater (*P* ≤ 0.05) caprylic, palmitic, stearic, saturated fatty acid (SFA) content and MUFA/PUFA ratio than other sources as well as higher total ω6 content than sources A and B but lower oleic, eicosanoic, linolenic, arachidonic; MUFA; EPA; UFA; total ω9 content and UFA/SFA ratio. Fatty acids pattern of sources A, B, and C showed almost similar values except for those of UFA, UFA/SFA, and total ω6 that showed value in favor of source C than those of sources A and B.

**Table 1:** Fatty acid profiles of whole edible egg parts (albumen + yolk) of different sources in retail market

Fatty acid % of total fatty acids^1^		Egg sources				Statistical analyses	
		A	B	C	D	RSME	*P*-value
Caprylic	C8:0	0.063^b^	0.038^b^	0.063^b^	0.968^a^	0.163	0.001
Capric	C10:0	0.070	0.038	0.063	0.065	0.034	0.559
Lauric	C12:0	0.065	0.038	0.063	0.053	0.029	0.462
Myristic	C14:0	0.410	0. 390	0.393	0.360	0.057	0.673
Palmitic	C16:0	25.74^b^	25.70^b^	25.11^b^	27.30^a^	0.505	0.001
Stearic	C18:0	9.08^b^	9.05^b^	9.17^b^	11.11^a^	0.419	0.001
Arachidic	C20:0	0.482	0.463	0.570	0. 560	0.066	0.092
Total saturated fatty acids		35. 92^b^	35.73^b^	35.41^b^	40.41^a^	0.802	0.001
Myristoleic	C14:1	0.133	0.193	0.147	0.173	0.080	0.739
Palmitoleic	C16:1	3.46	3.44	3.54	3.67	0.121	0.079
Oleic	C18:1	40.14^a^	39.12^a^	41.08^a^	34.38^b^	1.234	0.001
Eicosenoic	C20:1	0.344^a^	0.323^a^	0.354^a^	0.000^b^	0.081	0.002
Erucic	C22:1	0.066^a^	0.074^a^	0.118^a^	0.000^b^	0.059	0.098
Total monounsaturated fatty acids		44.14^a^	43.15^a^	45.24^a^	38.22^b^	1.249	0.001
Linoleic	C18:2	13.28	13.27	13.92	14.38	0.887	0.277
Linolenic	C18:3	0.413^a^	0.392^a^	0.378^a^	0.030^b^	0.059	0.001
Arachidonic	C20:4	1.661^a^	1.640^a^	1.623^a^	0.760^b^	0.085	0.001
Eicosapentaeonic, EPA	C20:5	0.066^a^	0.074^a^	0.072^a^	0.000^b^	0.033	0.027
Decohexaenoic, DHA	C22:6	0.436	0.416	0.319	0.668	0.134	0.078
Total polyunsaturated fatty acids		15.86	15.79	16.31	15.84	0.966	0.749
Total unsaturated fatty acids		59.99^b^	58.93^b^	61.68^a^	54.06^c^	1.150	0.001
UFA/SFA ratio		1.671^b^	1.648^b^	1.738^a^	1.338^c^	0.020	0.001
MUFA /PUFA ratio		1.36 ^b^	1.37 ^b^	1.36^b^	2.56^a^	0.112	0.001
Total n9		40.14^a^	39.13^a^	41.09^a^	34.38^b^	1.234	0.001
Total n6		14.94^b^	14.91^b^	15.54^a^	15.64^a^	0.925	0.001
Total n3		0.920	0.880	0.900	0.790	0.164	0.735
n6/n3 ratio		16.43	17.49	17.50	20.85	4.186	0.486

*SFA* saturated fatty acids, *UFA* unsaturated fatty acids, *PUFA* poly unsaturated fatty acids, *MUFA* mono unsaturated fatty acids, *RSME*, root square mean error

^1^Each value is 5 pooled samples per egg part

^abc^ Means within a row sharing common superscripts are significantly different

### Fatty acid profiles of the egg yolk

Data for fatty acid content of egg yolk are presented in Table 2. Differences among various sources of egg yolk were significant in majority of fatty acids, except for capric, lauric, myristic, arachidic, myristoleic, palmitoleic, and linoleic acids. In addition, variations among different sources of eggs in PUFA, UFA, total ω6, and ω3 content and ω6/ω3 fatty acid ratio were insignificant. Sources A, B, and C had lower (*P* ≤ 0.05) caprylic, stearic, erucic, linoleic, SFA and MUFA/PUFA ratio than those of source D but higher oleic, eicosanoic, linolenic, arachidonic, EPA, total ω9, MUFA and UFA/SFA ratio. There also were significant differences among sources A, B, and C in terms of palmitic, oleic, MUFA, DHA, SFA, and ω9 content, showing that source B had higher (*P* ≤ 0.05) values than those of sources A and C. Difference between the latter groups in palmitic acid and SFA content was also significantly in favor of source A.

**Table 2:** Fatty acid profiles of yolk of eggs of different sources in retail market

Fatty acid % of total fatty acids^1^		Egg sources				Statistical analyses	
		A	B	C	D	RSME	*P*-value
Caprylic	C8:0	0.063^b^	0.050^b^	0.063^b^	1.178^a^	0.222	0.001
Capric	C10:0	0.070	0.050	0.070	0.050	0.028	0.729
Lauric	C12:0	0.065	0.050	0.063	0.043	0.029	0.578
Myristic	C14:0	0.410	0. 390	0.393	0.420	0.031	0.873
Palmitic	C16:0	25.74^b^	26.73^a^	24.74^c^	27.08^a^	0.361	0.001
Stearic	C18:0	9.076^b^	9.060^b^	9.167^b^	11.19^a^	0.375	0.001
Arachidic	C20:0	0.472	0.463	0.470	0. 460	0.037	0.943
Total saturated fatty acids		35. 98^c^	36.82^b^	34.98^d^	40.41^a^	0.257	0.001
Myristoleic	C14:1	0.233	0.222	0.219	0.173	0.074	0.701
Palmitoleic	C16:1	3.553	3.542	3.539	3.516	0.183	0.991
Oleic	C18:1	40.23^b^	42.22^a^	40.22^b^	37.39^c^	0.590	0.001
Eicosenoic	C20:1	0.436^a^	0.426^a^	0.433^a^	0.000^b^	0.071	0.001
Erucic	C22:1	0.165 ^b^	0.153^b^	0.115^b^	0.492^a^	0.079	0.001
Total monounsaturated fatty acids		44.62^b^	46.56^a^	44.51^b^	41.57^c^	0.760	0.001
Linoleic	C18:2	13.29^b^	13.27^b^	14.35^b^	15.60^a^	1.798	0.267
Linolenic	C18:3	0.415^a^	0.405^a^	0.408^a^	0.000^b^	0.034	0.001
Arachidonic	C20:4	1.682^a^	1.663^a^	1.675^a^	0.908^b^	0.078	0.001
Eicosapentaeonic, EPA	C20:5	0.083^a^	0.065^a^	0.075^a^	0.000^b^	0.033	0.018
Decohexaenoic, DHA	C22:6	0.463^b^	0.553^a^	0.458^b^	0.468^b^	0.034	0.007
Total polyunsaturated fatty acids		15.93	15.96	16.96	16.98	1.876	0.758
Total unsaturated fatty acids		60.55	62.53	61.48	58.54	2.228	0.133
UFA/SFA ratio		1.687^a^	1.698^a^	1.757^a^	1.449^b^	0.006	0.001
MUFA /PUFA ratio		1.357^b^	1.343^b^	1.381^b^	2.124^a^	0.064	0.001
Total n9		40.23^b^	42.22^a^	40.22^b^	37.39^c^	0.590	0.001
Total n6		14.98	14.94	16.03	16.51	1.863	0.567
Total n3		0.960	1.030	0.940	0.870	0.100	0.209
n6/n3 ratio		15.72	14.64	17.22	18.99	2.452	0.125

*SFA* saturated fatty acids, *UFA* unsaturated fatty acids, *PUFA* poly unsaturated fatty acids, *MUFA* mono unsaturated fatty acids, *RSME*, root square mean error

^1^Each value is 5 pooled samples per egg part

^abc^ Means within a row sharing common superscripts are significantly different

### Lipid profiles of different edible parts of eggs

Data for fatty acid profiles of the whole edible parts of eggs and egg yolk are presented in Table 3. It should be mentioned that fatty acid patterns of yolk and whole edible parts (yolk + albumen) were similar. Differences among different parts of eggs were significant in case of most fatty acids, except for caprylic, capric, lauric, myristic, palmitic, stearic, myristoleic, palmitoleic, linoleic, linolenic, SFA, EPA, DHA, PUFA, total ω6, ω3 and ω6/ω3 ratio. Yolk exhibited greater concentrations (*P* ≤ 0.05) of oleic, eicosanoic, erucic, arachidonic, UFA, total ω9, UFA/SFA ratio, but lower arachidic acid and MUFA/PUFA ratio.

**Table 3:** Fatty acids contents of different egg parts in retail market

Fatty acid % of total fatty acids^1^		Egg parts		Statistical analyses	
		Whole eggs	Yolk	RSME	*P*-value
Caprylic	C8:0	0.283	0.399	0.197	0.424
Capric	C10:0	0.0587	0.0575	0.031	0.910
Lauric	C12:0	0.0556	0.0556	0.028	1.000
Myristic	C14:0	0.388	0.406	0.046	0.273
Palmitic	C16:0	25.96	26.07	0.434	0.483
Stearic	C18:0	9.600	9.628	0.303	0.795
Arachidic	C20:0	0.517^a^	0.464^b^	0.053	0.010
Total saturated fatty acids		36.86	37.03	0.599	0.453
Myristoleic	C14:1	0.162	0.210	0.078	0.093
Palmitoleic	C16:1	3.527	3.534	0.152	0.890
Oleic	C18:1	38.68^b^	40.01^a^	0.952	0.001
Eicosenoic	C20:1	0.253^b^	0.324^a^	0.076	0.015
Erucic	C22:1	0.064^b^	0.232^a^	0.069	0.001
Total monounsaturated fatty acids		42.69^b^	44.31^a^	1.012	0.001
Linoleic	C18:2	13.71	14.13	1.411	0.411
Linolenic	C18:3	0.302	0.307	0.047	0.768
Arachidonic	C20:4	1.42^b^	1.48^a^	0.082	0.045
Eicosapentaeonic, EPA	C20:5	0.053	0.056	0.033	0.831
Decohexaenoic, DHA	C22:6	0.495	0.485	0.096	0.771
Polyunsaturated fatty acids		15.98	16.46	1.486	0.373
Unsaturated fatty acids		58.67^b^	60.77^a^	1.764	0.003
UFA/SFA ratio		1.60^b^	1.65^a^	0.044	0.005
MUFA /PUFA ratio		1.66^a^	1.55^b^	0.089	0.002
Total n9		38.68^b^	40.01^a^	0.952	0.001
Total n6		15.26	15.61	1.455	0.497
Total n3		0.874	0.949	0.133	0.125
n6/n3 ratio		18.04	16.64	3.421	0.259

*SFA* saturated fatty acids, *UFA* unsaturated fatty acids, *PUFA* poly unsaturated fatty acids, *MUFA* mono unsaturated fatty acids, *RSME*, root square mean error

^1^Each value is 20 pooled samples per egg part

^ab^ Means within a row sharing common superscripts are significantly different

### Yolk lipids and cholesterols

Data for lipid profiles of the whole edible parts of eggs and egg yolk are presented in Table 4. Total lipid, total cholesterol, LDL, SFA/UFA ratio, and total antioxidant capacity were significantly higher for source D than those for the other sources but hypocholesterolemic index and malondialdehyde levels were lower. Difference in HDL was significantly showing higher values of sources C and D than those of A and B. Source A also showed higher HDL and malondialdehyde levels than source B and sources B and C, respectively. In addition, source B showed lower LDL levels than source C but higher SFA/UFA ratio.

**Table 4:** Lipids profile, saturated to unsaturated fatty acid ratio, malondialdhyde, total antioxidant capacity and indices of atherogenic and thrombogenic of eggs of different sources in retail market

Parameters^1^	Egg sources				Statistical analyses		RDA/day^2^
	A	B	C	D	RSME	*P*-value	
Yolk total lipid, g/100 g	31.87^ab^	29.94^b^	28.10^b^	33.10^a^	1.378	0.001	20-35, g
Saturated to unsaturated fatty acid ratio	0.598^b^	0.606^b^	0.574^c^	0.748^a^	0.011	0.001	----
Total cholesterol, mg/g yolk	14.8^ab^	15.9^c^	14.3^bc^	15.2^a^	0.781	0.005	300 mg
Yolk HDL, mg/g	6.91^b^	6.01^c^	7.76^a^	7.54^a^	0.237	0.001	NR
Yolk LDL, mg/g	3.39^bc^	3.22^c^	3.94^b^	4.64^a^	0.621	0.001	NR
Egg hypocholesterolaemic index	2.14^b^	2.10^b^	2.26^a^	1.82^c^	0.029	0.001	---
Egg malondialdhyde, μmol/l	12.91^a^	11.61^b^	12.11^b^	11.50^b^	0.746	0.001	---
Egg total antioxidants capacity, μmol/l	419^b^	417^b^	420^b^	425^a^	4.32	0.002	NR
Egg LDL/HDL ratio (Atherogenic index)^3^	0.489^b^	0.537^ab^	0.509^b^	0.615^a^	0.089	0.02	NR
Egg atherogenic index^4^	0.458^b^	0.463^b^	0.434^c^	0.533^a^	0.0098	0.001	---
Egg thrombogenic index	0.393^b^	0.397^b^	0.389^b^	0.784^a^	0.023	0.001	---

*LDL* low density lipoprotein, *HDL* high density lipoprotein, *NR* not reported, *RSME* root square mean error

^1^Each value is ten pooled samples per egg source used for determination of total lipids, total cholesterol, HDL, LDL, malondialdhyde and total antioxidant capacity analyses

^abc^ Means within a row sharing common superscripts are significantly different

^2^Dietary Reference Intakes (DRI) For Energy, Carbohydrate, Fiber, Fat, Fatty Acids, Cholesterol, Protein, and Amino Acids. Institute of Medicine (IOM). Standing Committee on the Scientific Evaluation of DietaryReference Intakes. 2002-2005. Available: http://www.nap.edu. Access May 2014.

^3^Atherogenic index was calculated according to Laudadio et al. [10] as the ratio of LDL-cholesterol to HDL-cholesterol

^4^Atherogenic index was calculated using the equation proposed by Ulbricht and Southgate (1991)

### Atherogenic and thrombogenic indices

Data for atherogenic and thrombogenic indices are shown in Table 4. It should be mentioned that atherogenic index calculated based on LDL/HDL ratio and that estimated using the fatty acid profiles of the eggs according to [11] were similar. These indicated that eggs of source D had higher atherogenic and thrombogenic indices than the other sources. In addition, atherogenic index calculated based on fatty acid profiles was the best for source C. Differences in atherogenic and thrombogenic indices were not significant among sources A, B, and C.

## Discussion

It was found that eggs in the retail market had different fatty acid and cholesterol profiles; lipid peroxidation biomarker; total antioxidant capacity; and hypocholesterolemic, atherogenic, and thrombogenic indices. Eggs from source D showed higher SFA and linoleic acid content and MUFA/PUFA ratio but lower MUFA, linolenic acid, EPA, DHA, and total ω9 content. On the other hand, total cholesterol content of source D was higher than that of sources B and C, and this concurred with higher LDL levels and LDL/HDL ratio and total lipids and total SFA (caprylic, palmitic, and stearic acids) content. In literature, high cholesterols corresponded with high total lipids and SFA content [7, 10, 33]. On the other hand, the low cholesterol of B source was coincided with lower SFA content and lower HDL and LDL levels. Egg consumption is negatively affected by lipid, cholesterol, and fatty acid profiles, and recently, by atherogenic and thrombogenic indices and their health implications [5, 8, 10] due to higher cardiovascular threat associated with increasing levels of blood cholesterol [9, 34]. However, the lipid and cholesterol contents of the present samples of eggs were found to be within the RDA for adult assuming daily consumption of one egg.

Differences in fatty acids profiles and cholesterols contents of eggs have been found to be affected by lipid metabolism and fats/fatty acids composition in the laying hen diets [4, 23, 25, 30, 34, 35]. However, there also are several other factors of second order that can affect lipid and cholesterol contents of eggs such as feed additives supplementation [6, 7, 24], age and strain of laying hens [28, 30], dietary fiber [6, 7, 33, 36], and flock husbandry [37] Cholesterol contents of eggs can also be manipulated by antioxidants supplementations [10].

Malondialdehyde, a lipid peroxidation biomarker, was higher in eggs from source A and this concurred with higher UFA and lower total antioxidant capacity of eggs. Increasing PUFA in chickens eggs is not total beneficial as it can lead to increasing lipid peroxidation and consequently oxidative rancidity if not accompanied with adequate supplementation of antioxidants [5, 7, 24]. Thus, increasing antioxidants supplementations for laying hens such as those of vitamin E, carotenoids, and Se could improve eggs keeping quality during storage and handling after harvest and thus increase costumers’ benefits. Free radicals formation during storage showed a negative effect on human health and welfare [7, 23, 38]. Thus, eggs enhanced with PUFA and antioxidants had a beneficial health benefits for human and recently recommended and showed increasing consumer preference [1, 7, 24]. Thus, the decrease in malondialdehyde of eggs from source D can be attributed partially to the high antioxidants contents, whereas the visa versa was shown by egg from source A. The latter effect was connected with high UFA/SFA ratio, which are highly susceptible to lipid peroxidation.

Eggs from source D had high of atherogenic and thrombogenic indices and this concurred with high LDL and SFA/UFA ratio. The atherogenic index (0.434–0.533), thrombogenic index (0.393–0781), and hypocholesterolemic index (1.81–2.26) of eggs of this study are comparable with those reported by [10, 12, 13]. A healthy diet was characterized by low hypercholesterolemic, atherogenic and thrombogenic indices [10, 12, 13]. It is well known that myristic and palmitic acids are among the most atherogenic agents, whereas stearic is thought to be neutral with respect to atherogenicity but is instead considered to be thrombogenic [14]. Eggs with high UFA content are preferable for customers due to low cholesterol (hypocholesterolemic), LDL/HDL and lower atherogenic index. However, eggs with greater PUFA are susceptible to peroxidation and thus enriching such eggs with antioxidants could decrease lipid peroxidation and improve quality of eggs in the retail market [10]. Similar to the present findings, [39] found that feeding atherogenic diet exhibited marked elevation in serum total cholesterol (hypercholesterolemic), LDL, VLDL, and triglycerides levels, along with decreased HDL levels. It should be considered that eggs with low atherogenic, thrombogenic and hypercholesterolemic indices are good for retarding atherosclerosis and thus risk of cardiovascular disorders [39], whereas eggs with low thrombogenicity decrease the threat of atrial fibrillation [15, 40].

## Conclusions

Eggs in the retail market in Jeddah city, Saudi Arabia, from May to August 2015 showed different fatty acid profiles, cholesterol profiles, malondialdehyde status, total antioxidant capacity and hypocholesterolemic, atherogenic, and thrombogenic indices. These variations can affect the nutritional values of eggs and hence customers health benefits. Thus, it can be suggested that standardization and quality control for eggs in the retail market based on fatty acid and cholesterol profiles can be introduced as a tool to reduce the risk of hypercholesterolemia, atherosclerosis, and thrombogenesis.

## Methods

### Material

Eggs were collected from four sources named A, B, C, and D, chosen randomly to represent different sources of eggs, in the retail market in Jeddah city, Saudi Arabia, from May to August 2015. A total number of 120 eggs of each source was used for egg quality determination as outlined in the first part of this research [5].

### Measurements

#### Fatty acids and lipid profiles of eggs

The sample size was 5 eggs/source/time, which was replicated four times, resulting in a total of 20 eggs/source. For total lipid and cholesterol profiles, total antioxidant capacity, and malondialdehyde determinations, the 20 samples were pooled over times for each source to represent 10 samples/source. A part of these samples were used for fatty acids analyses, for which the 10-pooled/source samples were pooled again to represent 5 samples/source.

### Yolk lipids and cholesterols

Lipid of the whole edible part of eggs (albumen + yolk) and yolk was extracted using the method given by [41], which includes homogenizing the yolk with 2:1 chloroform–methanol (v/v). Yolk cholesterols were determined using commercial diagnostic kits (Diamond Medical Services, Cairo, Egypt). Yolk total lipids [42], total cholesterol [43], high density lipoprotein (HDL) [44] were determined. LDL cholesterol levels were estimated using the equation [45]:

$$ \mathrm{L}\mathrm{D}\mathrm{L}\hbox{--} \mathrm{cholesterol} = \left(\mathrm{total}\ \mathrm{cholesterol} - \mathrm{H}\mathrm{D}\mathrm{L}\ \mathrm{cholesterol}\right) - \mathrm{triglycerides}/5. $$

The hypocholesterolemic index was calculated according to the equation [46]:

$$ \mathrm{Hypocholesterolemic}\ \mathrm{index} = \left(\mathrm{C}18:1 + \mathrm{C}18:2 + \mathrm{C}18:3 + \mathrm{C}20:3 + \mathrm{C}20:4 + \mathrm{C}20:5 + \mathrm{C}22:4 + \mathrm{C}22:6\right)/\left(\mathrm{C}14:0 + \mathrm{C}16:0\right) $$

### Fatty acids profile of egg edible parts and yolk

A part of the lipid was extracted from whole edible part of egg (yolk + albumen) and yolk was analyzed for its fatty acid contents by gas liquid chromatography (GLC) using Shimadzu Gas Chromatograph GC-4CM (PFE). A standard mixture of methyl esters was analyzed under identical conditions prior to running the samples. The instrument was equipped with flame ionization detector (FID) under the following conditions: an analytical glass column (3 × 3 mm i.d.) packed with 5 % diethylene glycol succinate on 80/100 Chromo Q. Operating temperature (°C) for column: 180 °C isothermal and injector and detector: 270 °C and gas flow rates (ml/min) for nitrogen: 30, hydrogen: 1, and air: 0.5. Chart speed 0.5 mm/min according to [47]. The retention times (t_R_) of the unknown sample of methyl esters was compared with those of the standard. The concentration of methyl esters was calculated by the triangulation method.

Total antioxidant capacity and malondialdehyde determinations for the whole edible of eggs were performed using diagnostic kits (Diamond Diagnostics, 33 Fiske St, Holliston MA 01746, USA), according to the method given by [48, 49], respectively.

Atherogenic and thrombotic indices:

Atherogenic and thrombogenic indices were calculated using the [11] equations as follows:

$$ \mathrm{Atherogenic}\ \mathrm{index}=\left(\mathrm{C}1\kern0.2em 2\kern0.34em :\kern0.32em 0\kern0.32em +\kern0.32em 4\kern0.42em \times \kern0.32em \mathrm{C}1\kern0.2em 4\kern0.34em :\kern0.32em 0\kern0.42em +\kern0.32em \mathrm{C}16\kern0.42em :\kern0.32em 0\right)\kern0.1em /\kern0.1em \left[\kern0.1em {\displaystyle \sum \kern0.2em \mathrm{M}\kern0.2em \mathrm{U}\mathrm{F}\kern0.1em \mathrm{A}+{\displaystyle \sum \left(\mathrm{n}\kern0.3em \hbox{-} \kern0.4em 6\right)\kern0.32em +{\displaystyle \sum \left(\mathrm{n}\kern0.3em \hbox{-} \kern0.4em 3\right)}}}\right] $$

$$ \begin{array}{l}\mathrm{Thrombogenic}\ \mathrm{index}=\left(\mathrm{C}1\kern0.2em 4\kern0.5em :\kern0.42em 0\kern0.5em +\kern0.5em \mathrm{C}16\kern0.6em :\kern0.5em 0\kern0.5em +\kern0.5em \mathrm{C}1\kern0.3em 8\kern0.5em :\kern0.5em 0\right)\kern0.1em /\left[0.5\right.\kern0.6em \times \kern0.4em {\displaystyle \sum \kern0.1em \mathrm{M}\kern0.2em \mathrm{U}\mathrm{F}\kern0.1em \mathrm{A}}\kern0.4em +\kern0.5em 0.5\kern0.5em \times \kern0.4em {\displaystyle \sum \left(\mathrm{n}\kern0.4em \hbox{-} \kern0.5em 6\right)}\kern0.4em +\kern0.4em 3\kern0.4em \times \\ {}{\displaystyle \sum \kern0.2em \left(\mathrm{n}\kern0.7em \hbox{-} \kern0.6em 3\kern0.2em \right)}\kern0.6em +\kern0.6em {\displaystyle \sum \kern0.2em \left(\mathrm{n}\kern0.7em \hbox{-} \kern0.7em 3\kern0.2em \right)\;}\kern0.1em /{\displaystyle \sum \kern0.2em \left(\mathrm{n}\kern0.7em \hbox{-} \kern0.8em 6\kern0.2em \right)}\kern0.1em \left.\kern0.1em \right]\end{array} $$

where MUFA is monounsaturated fatty acids.

### Statistical analysis

Analysis of variance was performed using straight run experimental design (one-way analyses of variance) of SAS software computer program [50] using the following model:

$$ \mathrm{Y}\mathrm{i}\mathrm{j} = \mu + \mathrm{Ai} + \mathrm{e}\mathrm{i}\mathrm{j} $$

μ = general mean, Ai: effect of egg source; eij: random error.

All percentages were transformed to arc sin to normalize data distribution before running the statistical analysis and Student–Newman–Keuls test to test mean differences if a significant probability value was obtained.
